# Hsa_circ_0001982 promotes the progression of breast cancer through miR-1287-5p/MUC19 axis under hypoxia

**DOI:** 10.1186/s12957-021-02273-8

**Published:** 2021-06-03

**Authors:** Zhimin Qiu, Ling Wang, Huaidong Liu

**Affiliations:** 1 Department of Breast Cancer, Jiangxi Cancer Hospital, Nanchang, Jiangxi China; 2 Department of Nursing, Jiangxi Health Vocational College, Nanchang, Jiangxi China; 3 Department of Oncology, Huaian Second People’s Hospital, The Affiliated Huaian Hospital of Xuzhou Medical University, No. 62 Huaihai South Road, Huai’an City, Jiangsu China

**Keywords:** Breast cancer, circ_0001982, miR-1287-5p, MUC19, Hypoxia

## Abstract

**Background:**

Breast cancer (BC) is the most commonly malignant tumor among women worldwide. Many studies have reported that circular RNAs (circRNAs) were participated in the regulation of multiple cancers development. However, the mechanism underlying hsa_circ_0001982 in breast cancer development is still unclear.

**Methods:**

Quantitative real-time polymerase chain reaction (qRT-PCR) was used to detect the levels of circ_0001982, microRNA-1287-5p (miR-1287-5p), and mucin 19 (MUC19) in BC tissues and cells under hypoxia. Moreover, glycolysis was evaluated by glucose consumption, lactic acid production, and hexokinase II (HK2) protein levels. The protein levels of cyclin D1, proliferating cell nuclear antigen (PCNA), and HK2 were determined by western blot assay. Cell proliferation, migration, and invasion were assessed by 3-(4,5-dimethyl-2-thiazolyl)-2,5-diphenyl-2-h-tetrazolium bromide (MTT) and transwell assays, respectively. The relationship between miR-1287-5p and circ_0001982 or MUC19 was predicted using starbase v3.0 or Targetscan, and verified by dual-luciferase reporter assay and RNA binding protein immunoprecipitation (RIP) assay. The xenograft model in nude mice was established to examine the effect of circ_0001982 in vivo.

**Results:**

The levels of circ_0001982 and MUC19 were upregulated, while miR-1287-5p was downregulated in BC tissues and cells under hypoxia. Knockdown of circ_0001982 hindered glycolysis, cell viability, migration, and invasion of BC cells under hypoxia. Mechanistic studies discovered that circ_0001982 could act as a sponge for miR-1287-5p to enhance MUC19 expression in BC cells. In addition, circ_0001982 silencing reduced xenograft tumor growth by regulating miR-1287-5p/MUC19 axis.

**Conclusion:**

Circ_0001982 affected BC cells glycolysis, proliferation, migration, and invasion through miR-1287-5p/MUC19 axis under hypoxia.

## Introduction

The epidemiological investigation shows that cancer caused vast numbers of deaths in women [1, 2]. Clinical breast cancer (BC) cases exhibit striking inheritance, epigenetic, and phenotypic diversity, which complicates the difficulty of BC research [3]. Although there is improvement in the surgery, radiotherapy, chemotherapy, and endocrine therapy of BC, the prognosis of BC patients is still unsatisfactory [4]. In addition, targeted therapy has significantly improved the survival rate of BC patients over the past 30 years, providing a new thinking for BC therapy [5]. Therefore, deep understanding of the molecular mechanism underlying BC progression is essential for developing targeted therapy.

As an important feature of solid tumors, hypoxia has been reported to induce BC progression, and enhance the risk of metastasis and death [6]. To enhance survival in a hypoxia environment, cancer cells undergo a so-called metabolic transformation, which with the hallmarks of the enhanced glycolysis and reduced oxidative phosphorylation, also termed the Warburg effect [7]. Warburg effects are conducive to the growth of cancer cells in the absence of oxygen [8]. Hexokinase 2 (HK2) is the first enzyme in cellular glycolytic metabolism, and also a vital rate-limiting enzyme [9]. In this study, we explored the pivotal molecule mechanism underlying the regulation of glucose consumption, lactate production and HK2 expression in BC cells.

Numerous evidence showed that circular RNAs (circRNAs) played essential roles in the pathogenesis of various cancers [10, 11]. Currently, circRNAs have been considered as promising therapeutic targets for BC [12]. For example, Yang et al*.* found that circAGFG1 induced cell proliferation, mobility, and invasion by sponging microRNA-195-5p (miR-195-5p) in triple-negative BC [13]. Moreover, Ren et al*.* reported that the downregulation of circDENND4C could repress glycolysis, migration, and invasion through interacting with smiR-200b/c in BC under hypoxia [14]. Interestingly, a newly discovered circRNA circ_0001982, which was located in chr1:173833394-17383618 and formed by the gene RNF111, has been confirmed to be upregulated in colorectal cancer [15]. Moreover, a recent research reported that circ_0001982 could serve as a carcinogenic regulator in BC by promoting proliferation and invasion. However, the role and regulatory mechanism of hsa_circ_0001982 in BC progression under hypoxia require in-depth research.

MicroRNAs (miRNAs) are small non-coding RNAs, about 22 nucleotides long, were reported to exert pivotal roles in the progression of human cancers, including BC [3, 16–18]. For instance, miR-944 inhibited the motility of colorectal cancer cells by modulating MET transcriptional regulator MACC1 [19]. MiR-16-5p restrained BC progression by targeting AKT serine/threonine kinase 3 (AKT3)/nuclear factor kappa B subunit 1 (NF-κB) signaling [20]. MiR-4732-5p contributed to BC development by regulating tetraspanin 13 (TSPAN13) [21]. Based on bioinformatics prediction, miR-1287-5p possesses the potential binding sites of circ_0001982, while MUC19 is a potential target of miR-1287-5p. MiR-1287-5p was reported to inhibit triple-negative BC growth [22]. Li et al*.* found that miR-593 suppressed BC cell proliferation and invasion through targeting mucin 19 (MUC19) [23]. The target relationship between miR-1287-5p and circ_0001982 or MUC19, as well as their functional association in regulating BC progression was explored.

This study aimed to investigate the biological function of circ_0001982 in BC and to determine its molecular mechanism by bioinformatics prediction and experimental verification.

## Materials and methods

### Tissue samples

BC tissues and adjacent normal tissues were obtained from 35 BC patients, who were recruited from Jiangxi Cancer Hospital, and stored at – 80 °C immediately. Informed written consent signed by all participants. Table 1 showed the clinicopathological characteristics of BC patients. Moreover, our research was conducted in accordance with the principles of the Declaration of Helsinki and was approved by the Ethics Committee of Jiangxi Cancer Hospital.

**Table 1:** The association between circ_0001982 expression and the clinicopathological characteristics of breast cancer patients

Parameter	Case	circ_0001982 expression		*P* value^a^
		Low (n = 17)	High (n = 18)	
Age (years)				0.6152
≤ 60	17	9	8	
> 60	18	8	10	
Menopause				0.1267
No	18	11	7	
Yes	17	6	11	
Tumor size				0.0041*
≤ 2 cm	16	12	4	
> 2 cm	19	5	14	
TNM stages				0.0003*
I–II	20	15	5	
III–IV	15	2	13	
Lymphatic metastasis				0.0284*
Negative	16	11	5	
Positive	19	6	13	

*TNM*, tumor-node-metastasis

*P < 0.05

^a^Chi-square test

### RNA isolation and quantitative real-time polymerase chain reaction (qRT-PCR)

Total RNA was collected from BC tissues and cells using the TRIzol reagent (Vazyme, Nanjing, China). Then, RNA was reverse transcribed to complementary DNA (cDNA) by PrimeScript™ RT Master Mix kit (Takara, Dalian, China) or miRNA 1st Strand cDNA Synthesis Kit (Vazyme). The qRT-PCR was performed by SYBR Green PCR Master Mix (Vazyme) and data was analyzed through the 2^−ΔΔCt^ method. Moreover, GAPDH and U6 were introduced as internal controls. Primers used in this research are as follows: circ_0001982 (forward 5′-ACAATCCAGCTGTTCCCTCA-3′, reverse 5′-GGTGCATCAGAAGGAATCTCA-3′), miR-1287-5p (forward 5′-GGGTGCTGGATCAGTGG-3′, reverse 5′-CAGTGCAGGGTCCGAGGTAT-3′), MUC19 (forward 5′-ATACCCCAGGATCAACGGGA-3′, reverse 5′-GTTGCCACGACAGTGGTTTC-3′), glyceraldehyde-3-phosphate dehydrogenase (GAPDH) (forward, 5′-GCTCTCTGCTCCTCCTGTTC-3′; reverse, 5′-ATCCGTTGACTCCGACCTTCAC-3′), and U6 (forward, 5′-CGCTTCGGCAGCACATATAC-3′, reverse, 5′-TTCACGAATTTGCGTGTCAT-3′).

### Cell culture, hypoxia stimulation, and transfection

Two BC cell lines (MDA-MB-231 and MDA-MB-468) and breast epithelial cells (MCF-10A) were brought from BeNa Culture Collection (Beijing, China). In our experiments, BC cells specifically refer to MDA-MB-231 and MDA-MB-468 cells. All cells were maintained in Dulbecco’s modified Eagle medium (DMEM; Invitrogen, Carlsbad, CA, USA) supplemented with 10% fetal bovine serum (FBS; Gibco, Carlsbad, CA, USA), penicillin (100 U/mL), and streptomycin (100 mg/mL) (Gibco) at 37 °C with 5% CO_2_. For hypoxia stimulation, BC cells were grown in a hypoxia chamber with 1% O_2_ at particular times (0, 3, 6, 12, 24, and 48 h).

The small interfering RNA against circ_0001982 (si-circ_0001982) and its negative control (si-NC), the inhibitor of miR-1287-5p (anti-miR-1287-5p) and its negative control (anti-miR-NC), and the small interfering RNA against-MUC19 (si-MUC19) were bought from Ribobio (Guangzhou, China). Lentivirus based short hairpin RNA for circ_0001982 (sh-circ_0001982) and negative control (sh-NC) were constructed by GeneCopoeia (Rockville, MD, USA). These oligonucleotides were transfected into the BC cells using the Lipofectamine 2000 reagent (Invitrogen) according to the manufacturer’s instructions.

### Subcellular fractionation location

In this assay, in order to detect the cellular localization of circ_0001982, BC cells were lysed in 200 μL Lysis Buffer (Life Technologies, Carlsbad, CA, USA), followed by centrifugation at 1300×*g* for 4 min. Transferring the supernatant containing cytoplasmic RNA, and the remaining liquid contains nuclear RNA. Subsequently, the supernatant and liquid were added buffer SK and absolute ethanol, followed by elution with column centrifugation. Finally, qRT-PCR analysis was applied to detect the cellular localization of circ_0001982, and U6 served as the nuclear control while GAPDH was used as cytoplasmic control.

### Cell viability, glucose consumption, and lactate production

Cell viability of BC cells transfected with si-circ_0001982 or si-NC were evaluated by cell counting kit-8 (CCK-8, Sigma-Aldrich, Shanghai, China) following the manufacturer’s instructions. The transfected cells were seeded in 96-well plates at a density of 1000 cells/well and incubated at 37 °C. The optical density was determined at 450 nm by a SpectraMax microtiter plate reader (Molecular Devices, Carlsbad, CA, USA) at 0, 12, 24, 48, and 72 h. Transfected or non-transfected BC cells (1 × 10^5^/well) were seeded into the 6-well plates overnight and then incubated under hypoxia or normal oxygen condition for 48 h. Glucose Assay Kit and Lactate Assay Kit (Sigma) were used for the evaluation of glucose consumption and lactate production, respectively. Glucose consumption and lactic acid production were normalized to the normal oxygen group.

### Western blot assay

Proteins were isolated by RIPA buffer (Vazyme), and Detergent Compatible Bradford Protein Quantification Kit (Vazyme) was used to determine the concentration of proteins. Subsequently, sodium dodecyl sulfate-polyacrylamide gel electrophoresis (SDS-PAGE) was used to separate the proteins and then the proteins were transferred onto the polyvinylidene difluoride (PVDF) membranes (Vazyme). After being blocked with 5% skimmed milk (Vazyme) and washed by phosphate-buffered saline (PBS), the membranes were incubated with corresponding primary antibodies: MUC19 (1:1000 dilution, ab212621, Abcam, Cambridge, UK), GAPDH (1:2000, ab37168, Abcam), PCNA (1:3000, Abways Technology, Inc., Shanghai, China), cyclin D1 (1:1000, Abways Technology), and antibodies against hexokinase II (HK2) (ab227198, 1:5000, 102 kDa) overnight at 4 °C. Then the membranes were incubated with the secondary antibody (1:3000, ab205718, Abcam) for 3 h. The blots were analyzed by the ChemiDoc™ MP Imaging System (Bio-Rad, Richmond, CA, USA) after being incubated with ECL kit (Vazyme).

### Cell migration and invasion assays

Cell migration and invasion abilities were carried out using a 24-well insert (8-μm pores, Corning Incorporated, Corning, NY, USA). For migration, BC cells re-suspended in DMEM medium with 10% FBS (100 μL) were plated into top chamber (non-coated membrane). For invasion, BC cells re-suspended in non-serum DMEM medium (100 μL) were placed into the top chamber with the Matrigel-coated membrane, while DMEM medium containing 10% FBS (600 μL) was supplemented into the bottom chamber. The cells (remaining on top chambers) were gently wiped using a cotton swab after incubation for 24 h. Then, the cells migrated or invaded to the bottom surfaces were fixed with 4% paraformaldehyde and subsequently stained with 0.1% crystal violet. Then, the cells were photographed and counted by a microscope (Leica, Solms, GER).

### Dual-luciferase reporter assay

Binding sites between miR-1287-5p and circ_0001982 or MUC19 were predicted by starBasev3.0 or targetscan, separately. The circ_0001982 or MUC19-3′UTR (3′-untranslated region) fragments containing putative and mutant circ_0001982 or MUC19 binding sites were synthesized and cloned into the pmirGlO luciferase reporter vector (Promega, Madison, WI, USA), namely circ_0001982-WT and MUC19-WT or circ_0001982-MUT and MUC19-MUT. The luciferase reporter plasmids (circ_0001982-WT, circ_0001982-MUT, MUC19-WT and MUC19-MUT) and miR-NC or miR-1287-5p were co-transfected into BC cells. At 48 h post-transfection, dual-luciferase reporter assay system (Promega) was employed to assess the luciferase activity, followed by normalizing with Renilla luciferase activity.

### Murine xenograft model in vivo

To investigate the role of circ_0001982 in vivo, stable MDA-MB-231 cells or control cells (MCF-10A) expressing sh-circ_0001982 or sh-NC were injected into the 5-week-old female nude mice (NU/NU Crl: NU-Fox1nu, Charles River Laboratories; Sulzfeld, Germany). In brief, 1 × 10^6^ cells were re-suspended in phosphate-buffered saline (PBS; 1:1 mixed with matrigel, Corning) and subcutaneously injected into the mammary fat pad of nude mice. MDA-MB-231 cells stable expressing sh-circ_0001982 were injected into the left mammary fat pad in a volume of 20 μL to NMRI: nu/nu mice while MCF-10A cells stable expressing sh-circ_0001982 were injected into the right mammary fat pad. Then, MDA-MB-231 and MCF-10A cells stable expressing sh-NC were injected into the mice in a same way. Tumor volume was measured every 7 days, and all mice were executed before tumors increased the diameter of 10 mm. Tumors were accumulated, and tumor volumes were computed by the equation: V (mm^3^) = (width)^2^ × length/2. The mice were executed after 5 weeks upon injection, and tumor tissues were weighed. Our research was carried out following guidelines of the national animal protection and ethics institute and was approved by the Animal Care and Use Committee of Jiangxi Cancer Hospital.

### Statistical analysis

Experimental data were assessed by GraphPad Prism (GraphPad, La Jolla, CA, USA) and presented as mean ± standard deviation (SD). The difference between two independent groups was analyzed by the Student’s *t*-test. The one-way analysis of variance (ANOVA) was used to evaluate the difference among three or more groups. Pearson’s correlation coefficient was utilized to analyze the correlation between hsa_circ_0001982 and miR-1287-5p in BC tissues. Each experiment was repeated at least three times independently. *P* < 0.05 represented statistical significant difference.

## Results

### Hypoxia treatment elevated circ_0001982 expression in a time-dependent manner

**Fig. 1:**
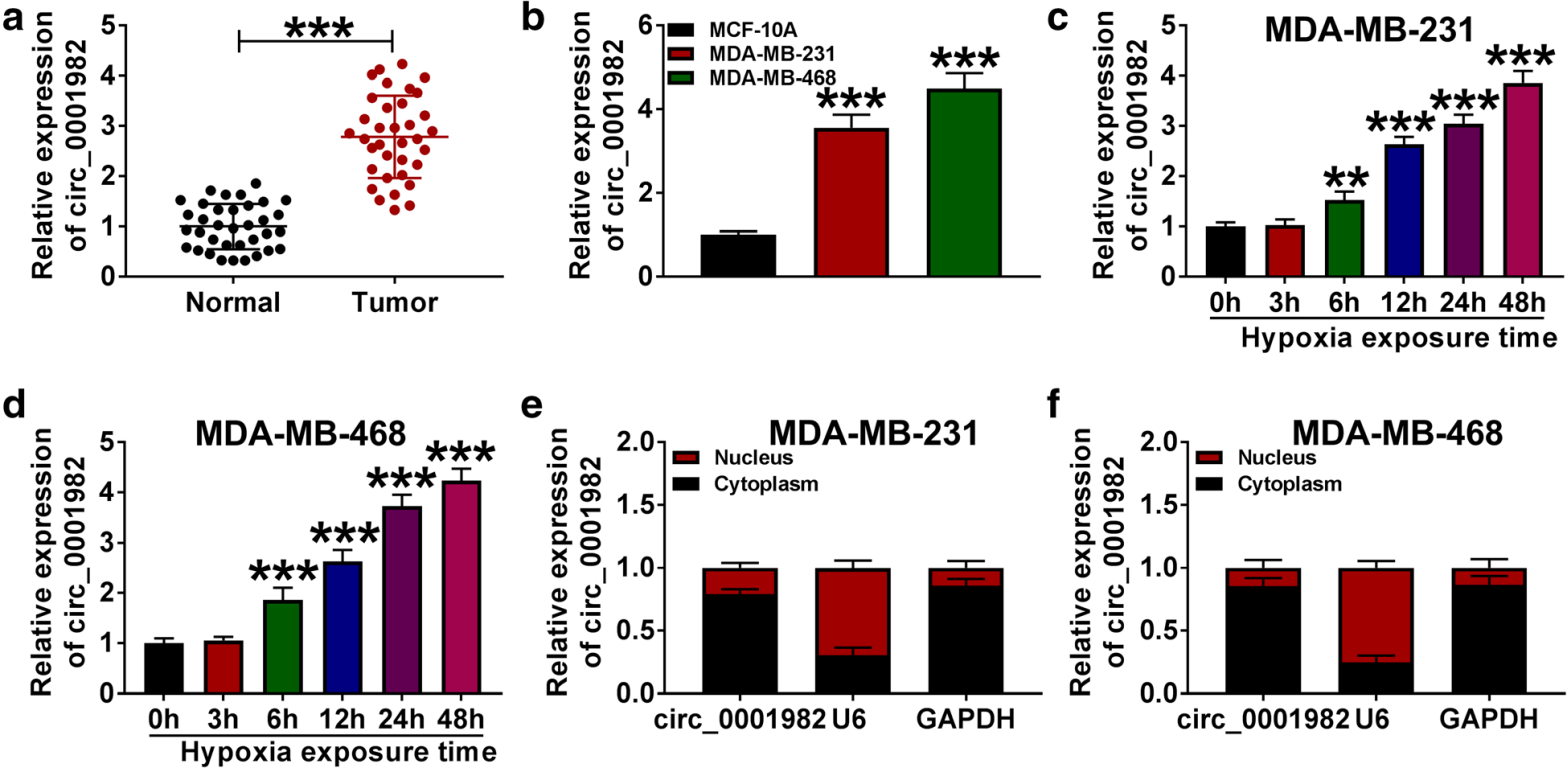
Hypoxia treatment elevated circ_0001982 expression in a time-dependent manner. **a** The expression of circ_0001982 in BC tissues (n = 35) and adjacent normal tissues (n = 35) was measured by qRT-PCR analysis. **b** The level of circ_0001982 in BC cells (MDA-MB-231 and MDA-MB-468) and breast epithelial cells (MCF-10A) was determined by qRT-PCR analysis. **c**, **d** QRT-PCR assay was conducted to evaluate the abundance of circ_0001982 in BC cells (MDA-MB-231 and MDA-MB-468) under hypoxia at time points (0 h, 3 h, 6 h, 12 h, 24 h, 48 h). **e**, **f** The cellular localization of circ_0001982 in MDA-MB-231 and MDA-MB-468 cells was analyzed by subcellular fractionation. *P < 0.05

The expression levels of circ_0001982 were detected by qRT-PCR in BC tissues, cells, normal tissues, and breast epithelial cells (MCF-10A). The results showed that circ_0001982 expression was upregulated in BC tissues compared to normal tissues (Fig. 1A). Besides, our data also verified that circ_0001982 expression was obviously increased in BC cell lines (MDA-MB-231 and MDA-MB-468) compared with breast epithelial cells (MCF-10A) (Fig. 1B). In addition, circ_0001982 expression in BC cells is gradually increased under hypoxia with time growth (0 h, 3 h, 6 h, 12 h, 24 h, 48h) (Fig. 1C, D). Then, we further explored the subcellular localization of circ_0001982 by using subcellular fractionation assay. As shown in Fig. 1E and F, circ_0001982 was mainly located in the cytoplasm of BC cells, suggesting that circ_0001982 might function in BC cells by posttranscriptional modification.

### Knockdown of circ_0001982 inhibited cell glycolysis, viability, migration, and invasion of BC cells under hypoxia

**Fig. 2:**
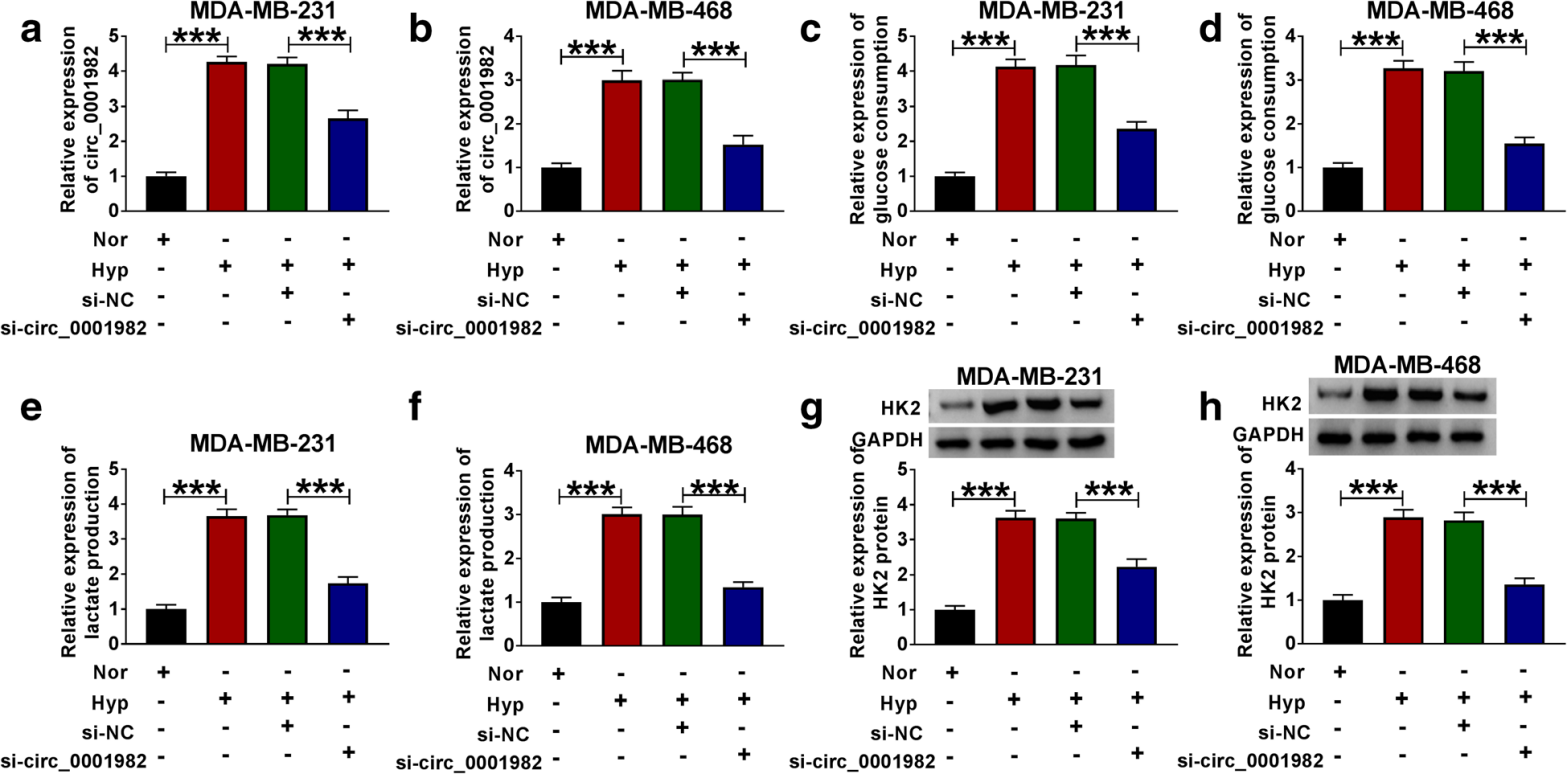
Circ_0001982 knockdown inhibited glycolysis in breast cancer cells under hypoxic conditions. MDA-MB-231 and MDA-MB-468 cells in hypoxic conditions were transfected with si-NC or si-circ_0001982. **a**, **b** QRT-PCR was used to detect the transfection efficiency of si-circ_0001982. The expression of circ_0001982 in BC cells (MDA-MB-231 and MDA-MB-468) under hypoxia conditions was detected. **c**, **d** The glucose consumption in MDA-MB-231 and MDA-MB-468 cells under 48 h of hypoxia treatment was assessed. **e**, **f** Lactic acid production in MDA-MB-231 and MDA-MB-468 cells under 48 h of hypoxia treatment was evaluated. **g**, **h** Western blot was used to detect the expression of HK2 in MDA-MB-231 and MDA-MB-468 cells under 48 h of hypoxia treatment. *P < 0.05

**Fig. 3:**
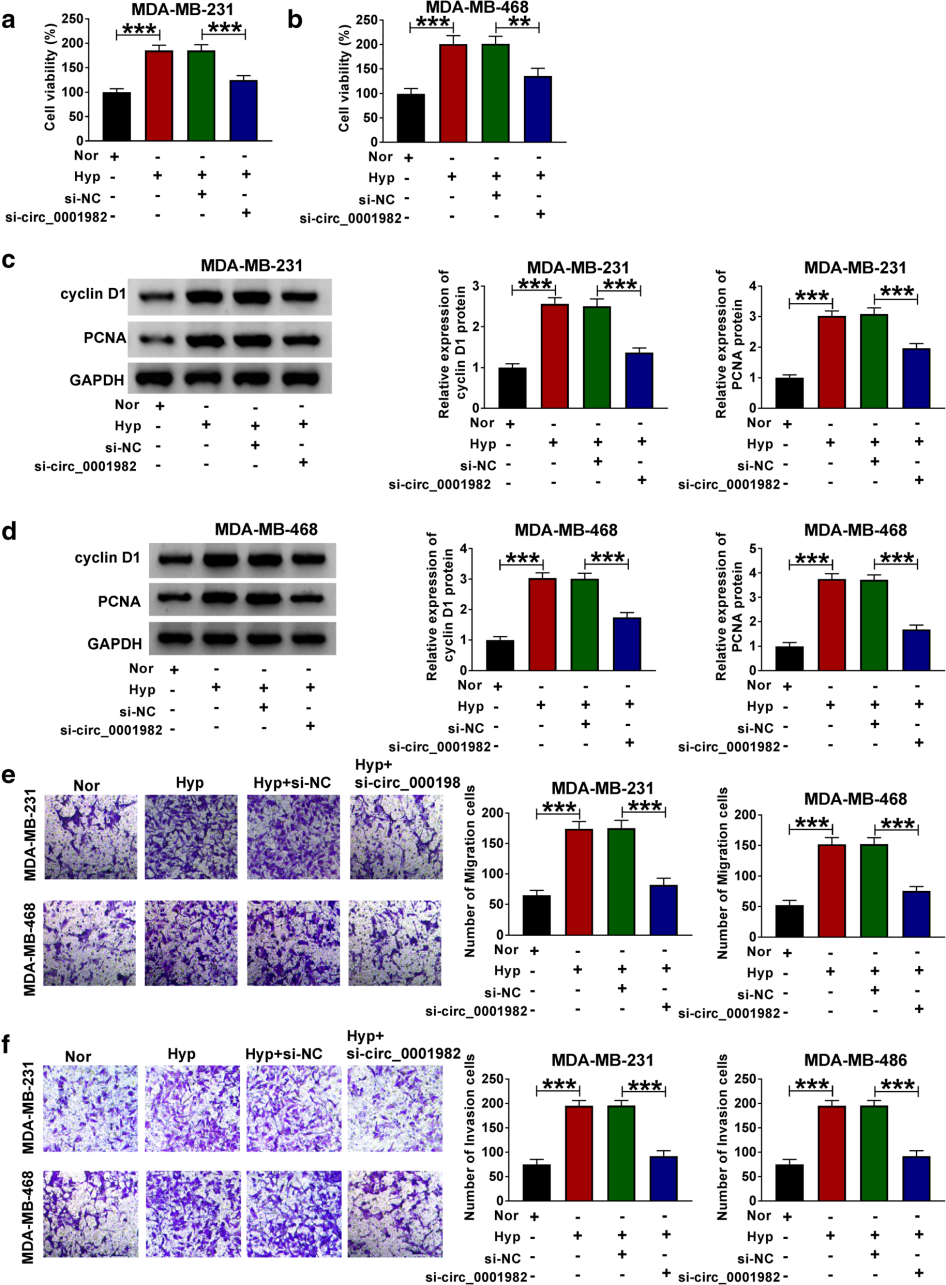
Knockdown of circ_0001982 inhibited breast cancer cell viability, migration, and invasion in hypoxic conditions. MDA-MB-231 and MDA-MB-468 cells were transfected with si-NC or si-circ_0001982. **a**, **b** MTT was used to detect cell viability activity of cell lines (MDA-MB-231 and MDA-MB-468) under 48 h of hypoxia treatment. **c**, **d** Western blot assay was applied to test the protein levels of Cyclin D1 and PCN1 in MDA-MB-231 and MDA-MB-468 cells under 48 h of hypoxia treatment. **e**, **f** Transwell assay was employed to explore MDA-MB-231 and MDA-MB-468 cells migration and invasion abilities under 48 h of hypoxia treatment. *P < 0.05

Next, we explored the function of circ_0001982 in BC cells by transfecting sh-circ_0001982 or sh-NC. The transfection efficiency was verified by qRT-PCR, and the expression of circ_0001982 in BC cells with hypoxia treatment was increased, and the knockdown of circ_0001982 reversed the elevated expression of circ_0001982 in BC cells (Fig. 2A, B). The data showed that after 48 h of hypoxia treatment, the glucose consumption of the BC cell line was increased, while circ_0001982 knockdown reduced glucose consumption (Fig. 2C, D). Besides, the lactic acid production of BC cell line increased significantly in BC cells with hypoxia treatment, while circ_0001982 knockdown reduced the production of lactic acid in BC cells with hypoxia treatment (Fig. 2E, F). Next, the HK2 protein level of the BC cells was increased significantly after 48 h of hypoxia treatment, while this tendency was reversed by knockdown of circ_0001982 (Fig. 2G, H). Forty-eight hours after hypoxia treatment, MTT was used to detect the activity of BC cells, and the data showed that circ_0001982 knockdown inhibited cell activity (Fig. 3A, B). Moreover, the expression of cyclin D1 and PCNA in BC cell lines was promoted by hypoxia treatment, and circ_0001982 knockdown reversed the expression of cyclin D1 and PCNA (Fig. 3C, D). Then, transwell assay was used to detect cell migration and invasion. After 48 h of hypoxia treatment, the data showed that migration and invasion of BC cell lines were promoted, while circ_0001982 knockdown inhibited cell migration and invasion (Fig. 3E, F).

### MiR-1287-5p inhibitor reversed circ_0001982 silencing-mediated effects on glycolysis, cell viability, migration, and invasion of BC cells

**Fig. 4:**
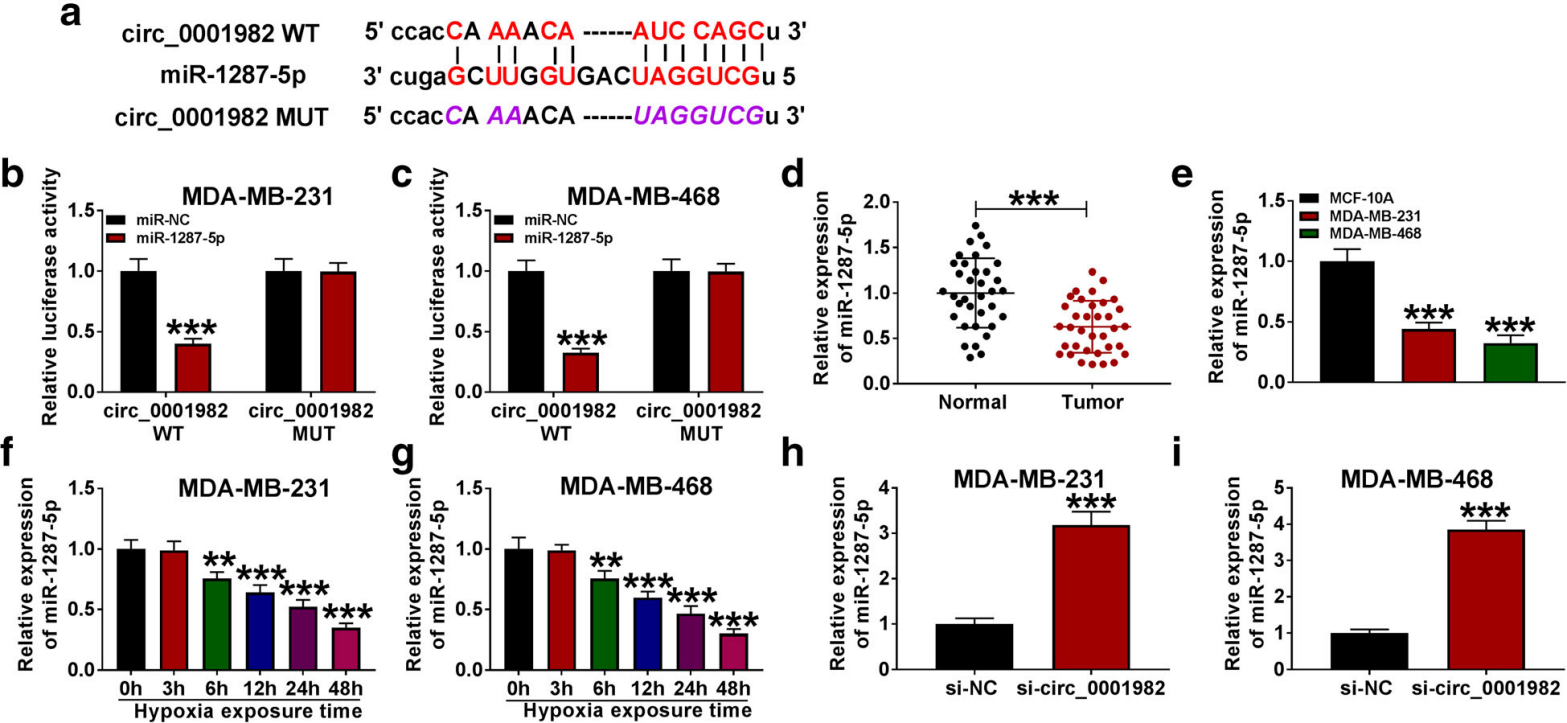
Circ_0001982 directly interacted with miR-1287-5p in BC cells. **a** StarBasev3.0 was utilized to predict the putative binding sites between circ_0001982 and miR-1287-5p. **b**, **c** The luciferase activity was measured in MDA-MB-231 and MDA-MB-468 cells co-transfected with circ_0001982-WT or circ_0001982-MUT and miR-138-5p or miR-NC. **d**, **e** The expression of miR-138-5p was measured by qRT-PCR in breast cancer tissues and cells. **f**, **g** The level of miR-1287-5p was determined using the qRT-PCR analysis in MDA-MB-231 and MDA-MB-468 cells under 48 h of hypoxia treatment. **h**, **i** The level of miR-1287-5p was determined using the qRT-PCR analysis in MDA-MB-231 and MDA-MB-468 transfected with si-NC or si-circ_0001982. *P < 0.05

To explore the mechanism underlying circ_0001982 in the progression of BC cells, we used StarBase v3.0 tools to predict the target sites of circ_0001982, and found that miR-1287-5p had a complementary binding sites with circ_0001982 (Fig. 4A). Through dual-luciferase reporter assay, we found that miR-1287-5p overexpression remarkably reduced the luciferase activity of circ_0001982 WT group, but not circ_0001982 MUT group (Fig. 4B, C), which confirmed the target relationship between circ_0001982 and miR-1287-5p. Besides, miR-1287-5p is lower expressed in BC tissues and BC cells in contrast with adjacent normal tissues and MCF-10A cells (Fig. 4D, E). After 48 h of hypoxia treatment, the expression of miR-1287-5p was gradually decreased in BC cell lines with time increases (Fig. 4F, G). Furthermore, the expression of miR-1287-5p was elevated in BC cells with circ_0001982 knockdown (Fig. 4H, I).

**Fig. 5:**
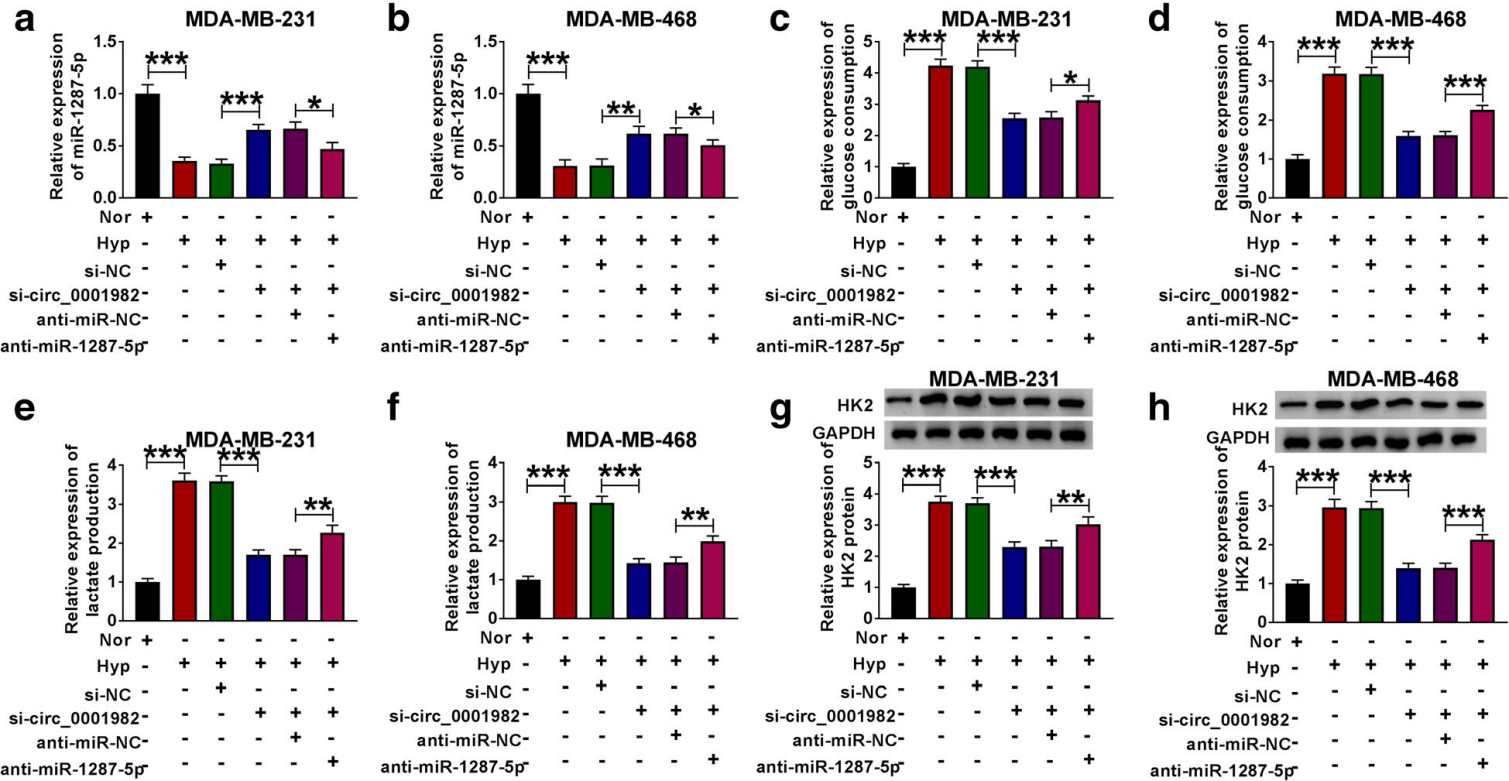
Downregulation of miR-1287-5p reversed the inhibitory effects of circ_0001982 knockdown on progression of BC cells. **a**–**h** MDA-MB-231 and MDA-MB-468 cells in normal and hypoxia conditions were transfected with si-NC, or si-circ_0001982, si-circ_0001982 + anti-miR-NC, or si-circ_0001982 + anti-miR-1287-5p, respectively. **a**, **b** The expression of miR-1287-5p and the transfection efficiency was evaluated using qRT-PCR. **c**, **d** The glucose consumption in the transfected BC cells was detected. **e**, **f** The lactic acid production in MDA-MB-231 and MDA-MB-468 cells under 48 h of hypoxia treatment was evaluated. **g**, **h** Western blot assay was used to detect the protein level of HK2 in MDA-MB-231 and MDA-MB-468 cells under 48 h of hypoxia treatment. *P < 0.05

**Fig. 6:**
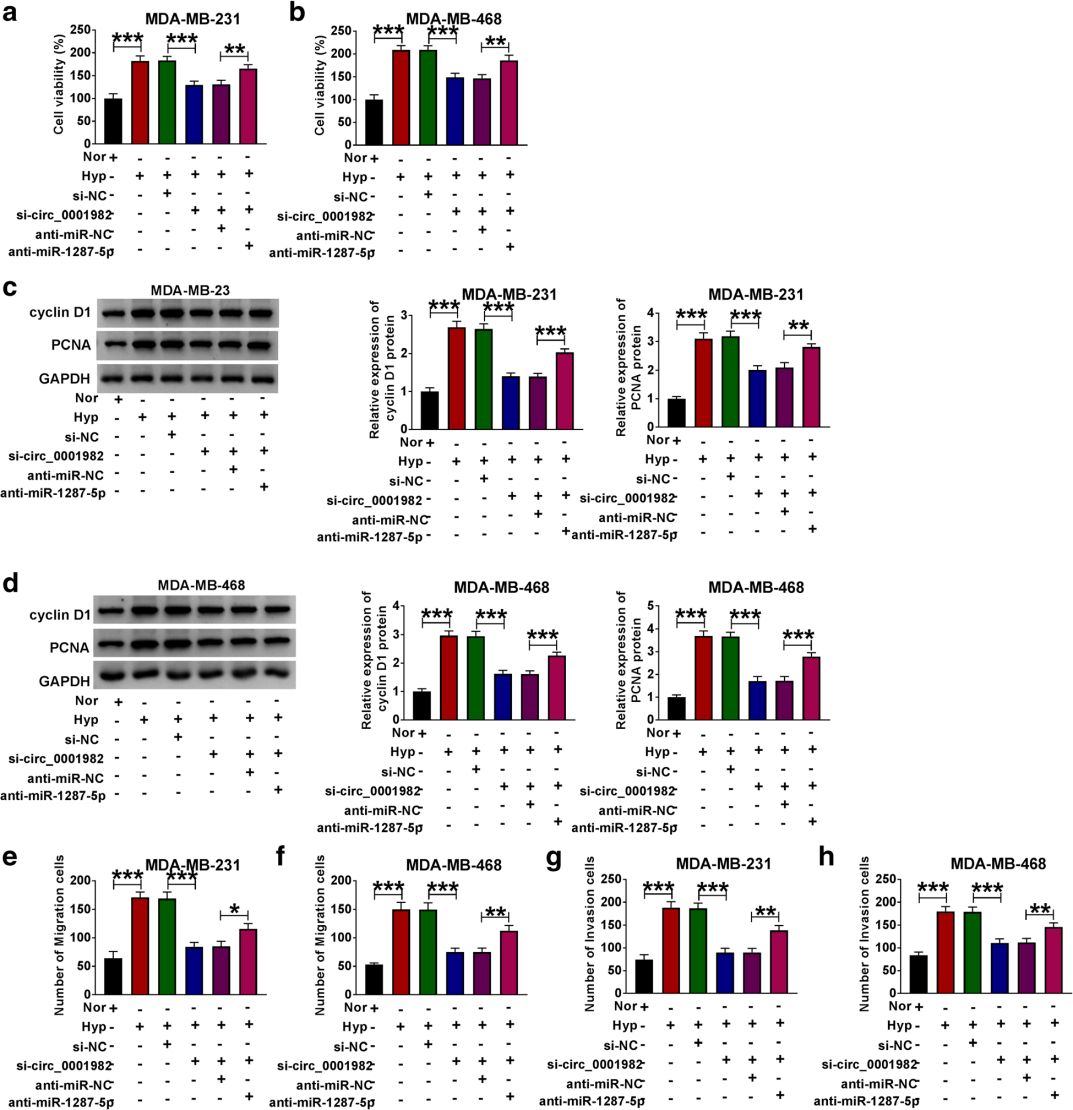
Downregulation of miR-1287-5p reversed the inhibitory effects of circ_0001982 knockdown on cell viability, migration, and invasion on progression of BC cells. The MDA-MB-231 and MDA-MB-468 cells in normal and hypoxia conditions were transfected with si-NC, or si-circ_0001982, si-circ_0001982 + anti-miR-NC, or si-circ_0001982 + anti-miR-1287-5p. **a**, **b** Cell activity was assessed by MTT assay in MDA-MB-231 and MDA-MB-468 cells under 48 h of hypoxia treatment. **c**, **d** The protein levels of cyclin D1 and PCNA in transfected cells were detected by western blot. **e**–**h** Transwell assay was used to investigate the migration and invasion capacities of MDA-MB-231 and MDA-MB-468 cells upon transfection. *P < 0.05

Besides, we further investigated the functional effects of miR-1287-5p in BC. The expression of circ_0001982 in normal conditions was significantly decreased compared with the cells in hypoxia conditions. And si-circ_0001982 upregulated the expression of miR-1287-5p in hypoxia conditions, while co-transfection of anti-miR-1287-5p partly reversed this effect (Fig. 5A, B). Besides, the BC cells consumed more glucose in hypoxia conditions than the cells which were in normoxia. Moreover, knockdown of circ_0001982 inhibited the consumption of glucose in BC cells under hypoxia conditions, while downregulation of miR-1287-5p partly reversed the suppression effect of si-circ_0001982 on the consumption of glucose in hypoxia conditions (Fig. 5C, D). Similarly, circ_0001982 knockdown suppressed the elevated lactic acid production (Fig. 5E, F) and HK2 protein level (Fig. 5G, H) that induced by hypoxia treatment, while miR-1287-5p inhibitor partly reversed the effects of circ_0001982 knockdown in BC cells under hypoxia treatment. Furthermore, the BC cell viability was enhanced by hypoxia treatment, and miR-1287-5p inhibition elevated the downregulated viability of cells that induced by si-circ_0001982 (Fig. 6A, B). Moreover, miR-1287-5p inhibition also reversed the suppression effects on the protein levels of cyclin D1 and PCNA in BC cells with 48 h of hypoxia treatment (Fig. 6C, D). In addition, the migration and invasion abilities of BC cells with hypoxia treatment were stronger than that under normoxia conditions. Circ_0001982 knockdown inhibited cell migration and invasion, but these effects were inhibited by miR-1287-5p inhibition (Fig. 6E–H).

### MiR-1287-5p interacted with MUC19 in BC cells

**Fig. 7:**
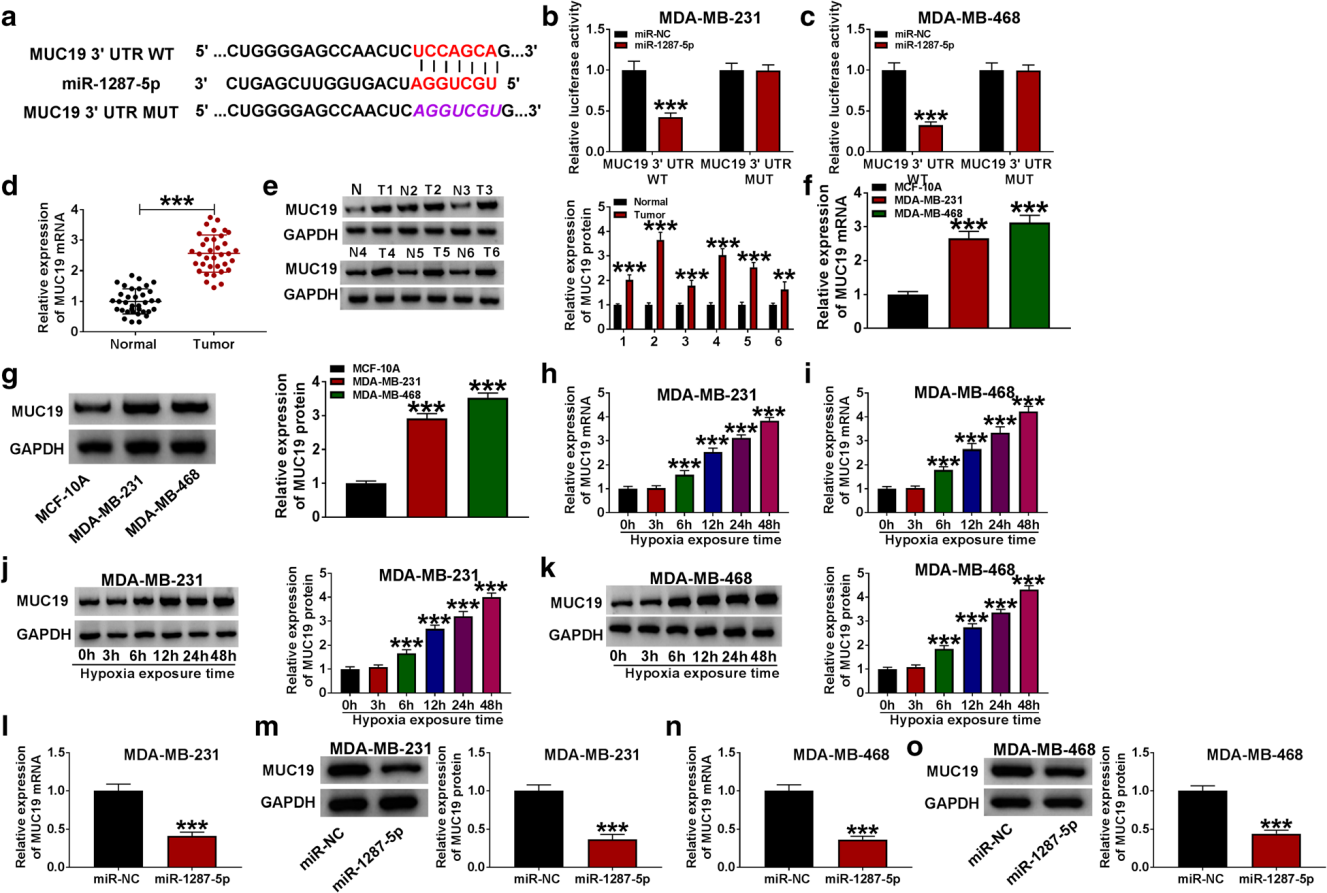
MiR-1287-5p directly interacted with MUC19 in BC cells. **a** Targetscan predicted the putative binding sites between circ_0001982 and miR-1287-5p. **b**, **c** The BC cells were co-transfected with MUC19 3′UTR-WT + miR-NC, MUC19 3′UTR-WT + miR-1287-5p, MUC19 3′UTR-MUT + miR-NC, and MUC19 3′UTR-MUT + miR-1287-5p. And the luciferase activity was measured. **d**–**g** The mRNA and protein levels of MUC19 were measured by RT-PCR and western blot in breast tissues and cells. **h**–**k** The mRNA and protein levels of MUC19 were measured by qRT-PCR and western blot assays in breast MDA-MB-231 and MDA-MB-468 cells under 48 h of hypoxia treatment. **l**–**o** The level of MUC19 was determined using the qRT-PCR and western blot analysis in MDA-MB-231 and MDA-MB-468 transfected with miR-1287-5p mimic under 48 h of hypoxia treatment. *P < 0.05

Binding sites between miR-1287-5p and MUC19 were predicted by TargetScan (Fig. 7A). And this target relationship was verified by dual-luciferase reporter assay, as identified by the decreasing luciferase activity in miR-1287-5p and MUC19 3′UTR co-transfected group (Fig. 7B, C), indicating that miR-1287-5p could sponge MUC19. Besides, MUC19 was highly expressed in BC tissues (Fig. 7D, E) and cells (Fig. 7F, G) than that in healthy tissues and MCF-10A cells. Furthermore, qRT-PCR and western blot assays showed that the levels of MUC19 mRNA and protein in BC cells were gradually increased with the time growth (within 48 h) under hypoxia treatment (Fig. 7H–K). Additionally, inhibition of miR-1287-5p decreased the mRNA and protein levels of MUC19 in BC cell lines (Fig. 7L–O).

### MUC19 overexpression overturned miR-1287-5p mimic-mediated effects on BC cells in hypoxia conditions

**Fig. 8:**
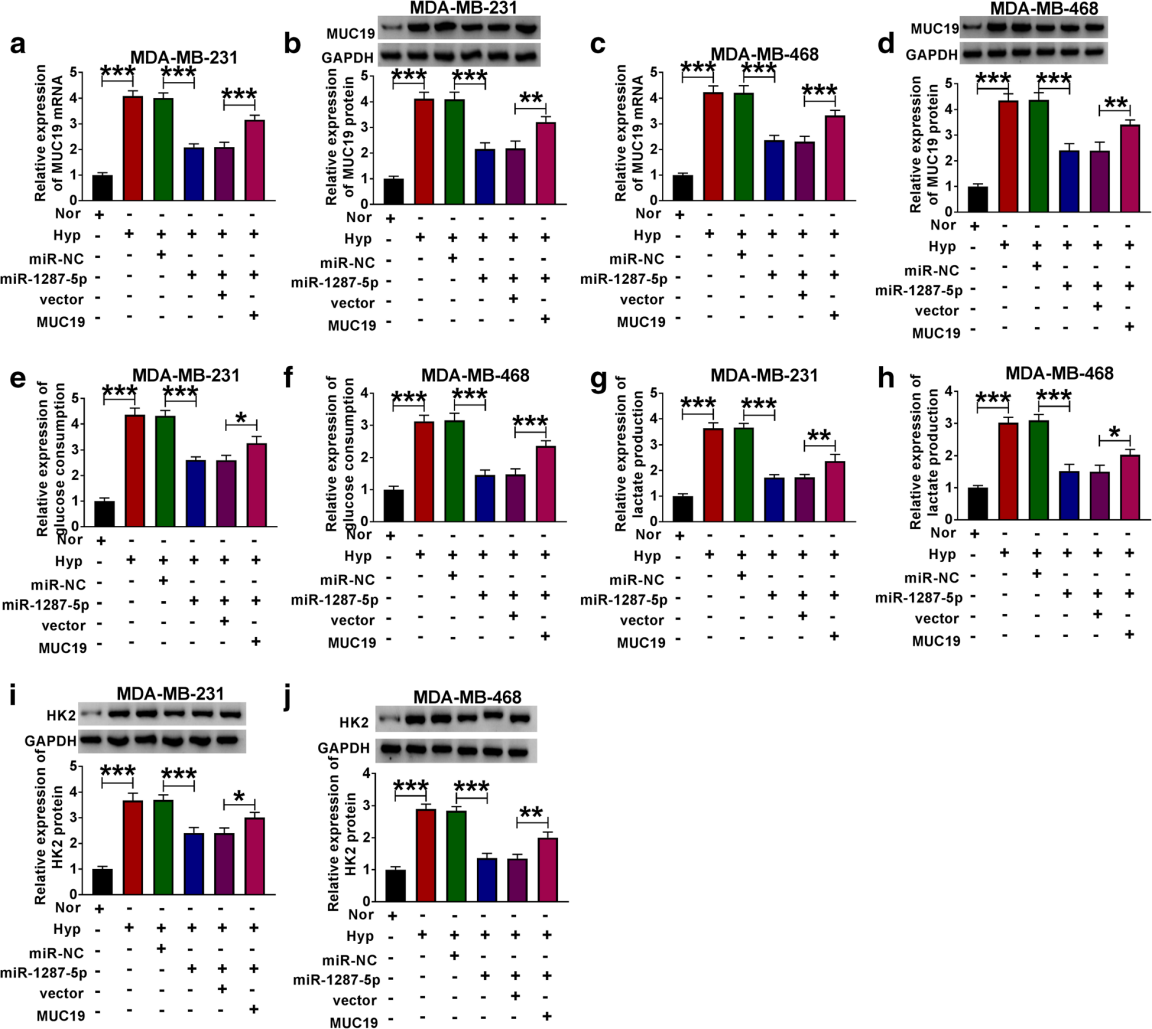
MUC19 overexpression partly reversed the inhibitory effects of miR-1287-5p mimic on BC cells glycolysis. **a**–**d** The mRNA and protein levels of MUC19 in BC cells co-transfected with miR-NC, miR-1287-5p, miR-1287-5p + vector, or miR-1287-5p + MUC19 were evaluated by qRT-PCR in MDA-MB-231 and MDA-MB-468 cells under 48 h of hypoxia treatment. **e**, **f** Glucose consumption in MDA-MB-231 and MDA-MB-468 cells under 48 h of hypoxia treatment was assessed. **g**, **h** Lactic acid production in MDA-MB-231 and MDA-MB-468 cells under 48 h of hypoxia treatment was detected. **i**, **j** Western blot assay was used to detect the protein level of HK2 in MDA-MB-231 and MDA-MB-468 cells under 48 h of hypoxia treatment. *P < 0.05

**Fig. 9:**
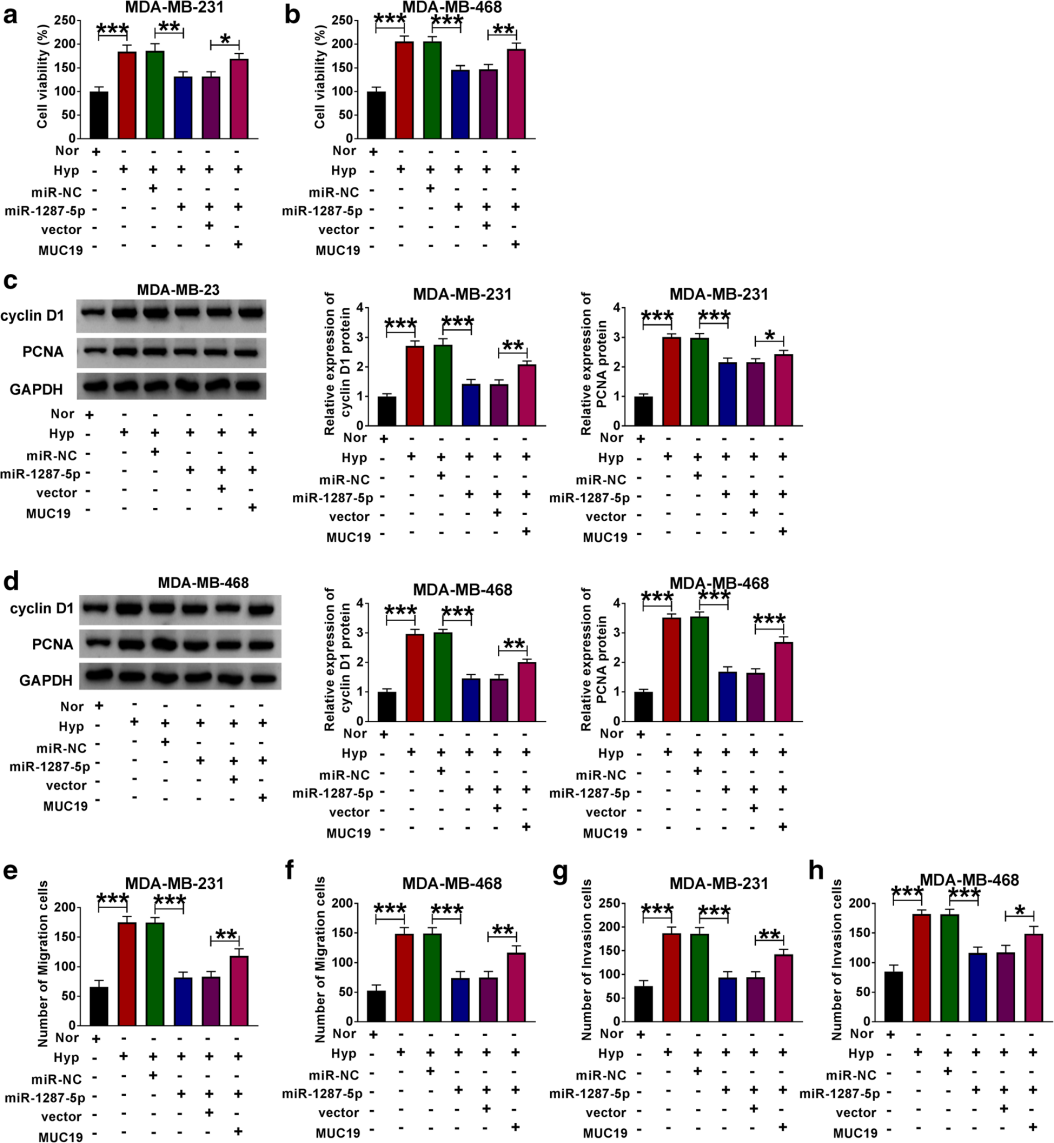
MUC19 overexpression partly reversed the inhibitory effects of miR-1287-5p mimic on BC cell viability, migration, and invasion. **a**–**h** The MDA-MB-231 and MDA-MB-468 cells under 48 h of hypoxia treatment were co-transfected with miR-NC, miR-1287-5p, miR-1287-5p + vector, or miR-1287-5p + MUC19, respectively. **a**, **b** Cell activity was assessed by MTT assay in transfected MDA-MB-231 and MDA-MB-468 cells under 48 h of hypoxia treatment. **c**, **d** The protein levels of cyclin D1 and PCNA in transfected MDA-MB-231 and MDA-MB-468 cells under 48 h of hypoxia treatment were detected by western blot. **e**–**h** Transwell assay was used to investigate the migration and invasion abilities of transfected MDA-MB-231 and MDA-MB-468 cells under 48 h of hypoxia treatment. *P < 0.05

To investigate whether MUC19 is involved in miR-1287-5p-mediated BC progression, BC cells in normal or hypoxia conditions were transfected with miR-NC, miR-1287-5p, miR-1287-5p + vector, or miR-1287-5p + MUC19, respectively. As shown in Fig. 8A–D, MUC19 overexpression partly reversed the suppression effect of miR-1287-5p mimic on cell viability in BC cells under hypoxia treatment. And miR-1287-5p mimic inhibited glucose consumption, lactate production, and HK2 protein level in BC cells under hypoxia conditions, while these effects were partly reversed by the co-transfection of MUC19 overexpression (Fig. 8E–J). In addition, miR-1287-5p deficiency weakened the suppressive effects of MUC19 knockdown on cell viability, migration, and invasion in cells with hypoxia treatment (Fig. 9A–H).

### Circ_0001982 regulated MUC19 via miR-1287-5p to mediate xenograft tumor growth

**Fig. 10:**
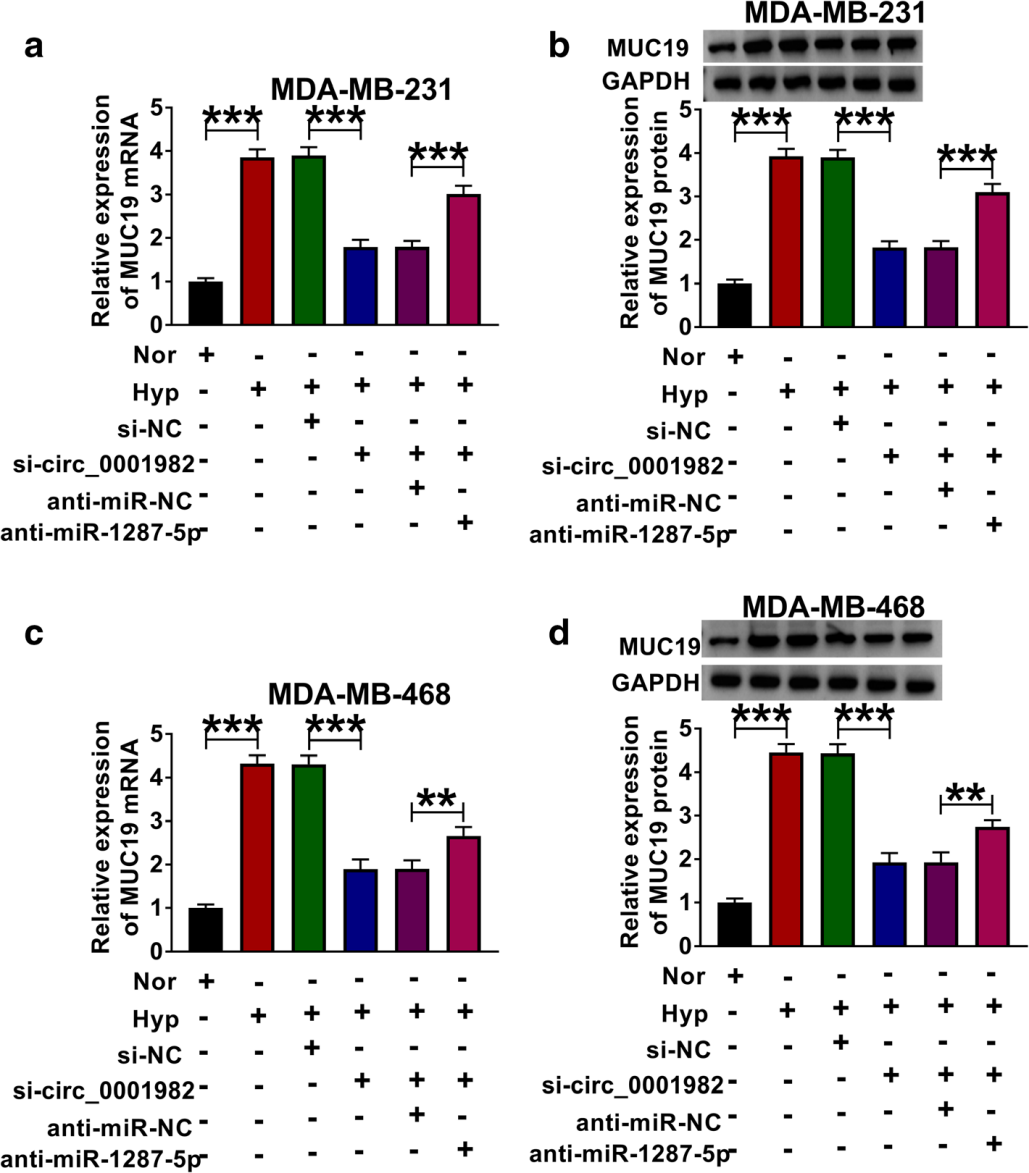
Circ_0001982 regulated MUC19 expression by miR-1287-5p. The MDA-MB-231 and MDA-MB-468 cells in normal and hypoxia conditions were co-transfected with si-NC, si-circ_0001982, si-circ_0001982 + anti-miR-NC, or si-circ_0001982 + anti-miR-1287-5p, respectively. **a**–**d** The mRNA and protein levels of NUC19 were evaluated using qRT-PCR and western blot in MDA-MB-231 and MDA-MB-468 cells upon transfection. *P < 0.05

**Fig. 11:**
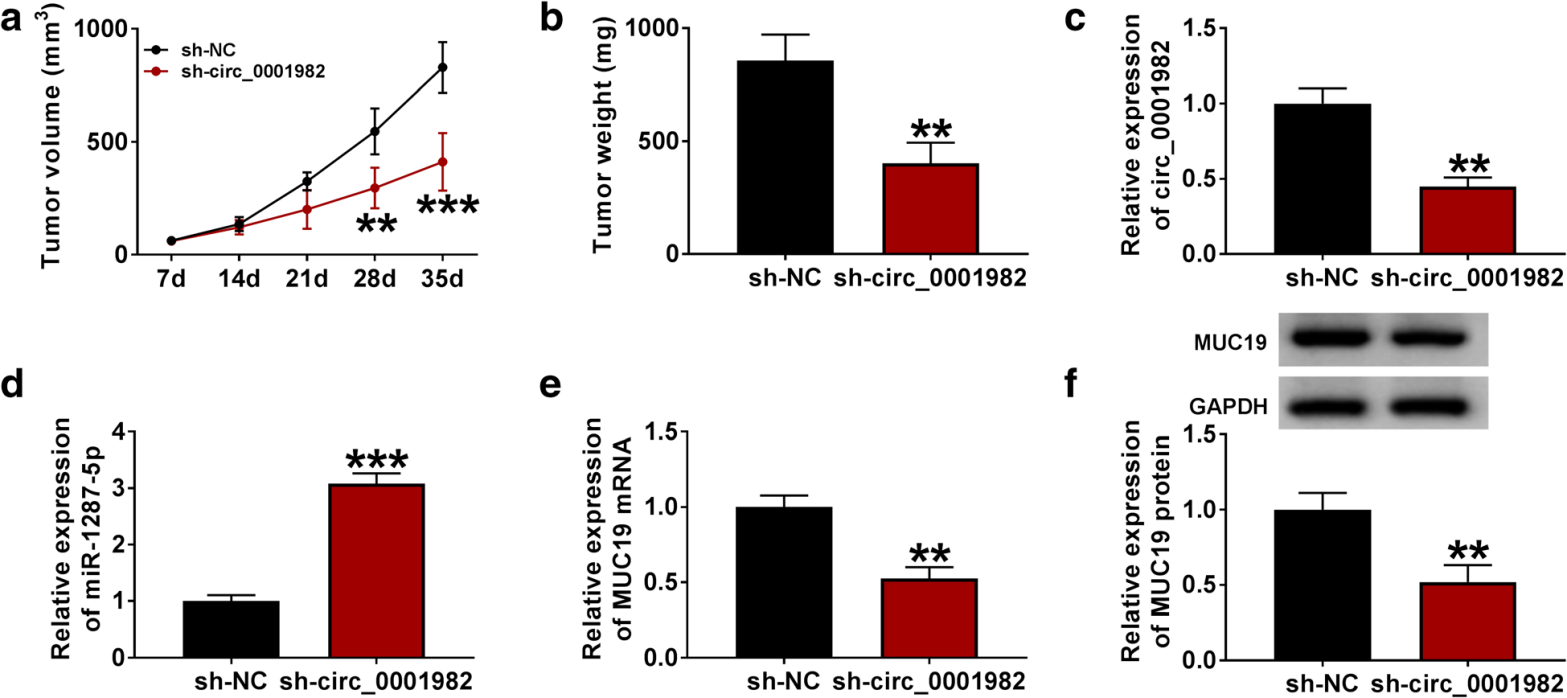
Circ_0001982 knockdown inhibited tumor growth in vivo. The xenograft tumor model in nude mice was established by injecting stable MDA-MB-231 cells expressing sh-circ_0001982 or sh-NC. **a** Tumor volume and growth curves of xenograft tumors were established. **b** The weight of xenograft tumor tissues. **c**, **d** The mRNA levels of circ_0001982 and MUC19 in sh-circ_0001982 or sh-NC group were quantified by qRT-PCR. **e**, **f** The mRNA and protein levels of MUC19 in xenograft tumors were evaluated. *P < 0.05

In order to investigate the regulation effect of circ_0001982 on MUC19, the BC cells were transfected with si-NC, si-circ_0001982, si-circ_0001982 + anti-miR-NC, or si-circ_0001982 + anti-miR-1287-5p, respectively. The results suggested that circ_0001982 knockdown reversed the elevated mRNA and protein levels of MUC19 in BC cells in hypoxia conditions, and miR-1287-5p inhibitor partly reversed the effects of si-circ_0001982 on MUC19 mRNA and protein levels, suggesting that circ_0001982 upregulated MUC19 expression by sponging miR-1287-5p (Fig. 10A–D). To investigate the anti-tumor effect of circ_0001982 silence in vivo, MDA-MB-231 and MCF-10A cells stably transfected with sh-circ_0001982 or sh-NC were used to establish xenograft model in vivo. After injection for 5 weeks, the mice were euthanized. As displayed in Fig. 11A and B, tumor volume and weight were decreased in sh-circ_0001982 group compared to the sh-NC group. Besides, circ_0001982 expression was notably downregulated in the sh-circ_0001982 group compared to sh-NC group (Fig. 11C). Moreover, the expression of miR-1287-5p was increased in the sh-circ_0001982 group in contrast with the sh-NC group (Fig. 11D). However, the mRNA and protein levels of MUC19 were decreased in the sh-circ_0001982 group compared to sh-NC group (Fig. 11E, F).

## Discussion

As one of the most common malignant tumors and the main cause of cancer-related death in women, the prognosis of BC patients is still poor. Hypoxia is a key feature of cancers, and circRNAs have been found to participate in the regulation of cancer cells malignant phenotypes upon hypoxia [24]. Many reports have shown that circRNAs are dysregulated in many cancers, and circRNAs could regulate BC progression in hypoxia conditions [14]. In our research, we found that circ_0001982 promoted breast cancer progression through mediating miR-1287-5p/MUC19 pathway.

CircRNAs regulate multiple human cancer-related genes, such as oncogenes and suppressor genes [25]. For example, circ_0021977 suppressed colorectal cancer cell proliferation by targeting miR-10b-5p/P21 and P53 axis [26]. Circular RNA CDR1as enhanced E2F3 stability and promoted the growth of nasopharyngeal carcinoma by sponging miR-7-5p [27]. Besides, NUDT21 participated in the occurrence of liver cancer [28]. Collectively, circRNAs acted as major modulators in tumor progression [29]. Many studies have demonstrated that circ_0001982 was overexpressed in human cancers and it was closely related to tumor progression. For instance, Tang et al*.* found that circ_0001982 promoted BC cell carcinogenesis [30]. Additionally, our data indicated that circ_0001982 knockdown blocked cell glycolytic metabolism, cell viability, migration, and invasion of BC cells under hypoxia conditions. Our data proved that circ_0001982 acted as a carcinogenesis factor to promote the progression of BC, which is in consistent with its function in colorectal cancer [15].

It was well known that circRNAs could act as ceRNAs to modulate gene expression via sponging miRNAs in multiple cancers. MicroRNAs (miRNAs) are short non-coding RNAs, which regulate gene expression by binding to the 3′UTR of target mRNAs. Recent research has disclosed that the dysregulation of miRNAs was closely associated with cancer progression [31, 32]. Khandelwal et al*.* reported that microRNA-590-5p could be used as a fluid biopsy marker for non-small cell lung cancer [33]. In present research, circ_0001982 was mainly located in cytoplasm, which indicated that circ_0001982 may function as a ceRNA in BC cells. Then, StarBase v3.0 was employed to predict the target of circ_0001982, and miR-1287-5p was predicted to contain the complementary binding sites of circ_0001982. MiR-1287-5p has been reported to function as a tumor suppressor to inhibit BC progression [22, 34]. Here, we found that miR-1287-5p level was reduced in BC tissues and cells, and miR-1287-5p expression was negatively correlated with circ_0001982 expression. In addition, we found that miR-1287-5p overexpression suppressed the malignant behaviors of BC cells, and miR-138-5p knockdown reversed circ_0001982 silencing-mediated effects on BC cells. These findings revealed that circ_0001982 contributed to BC progression by sponging miR-1287-5p in BC cells.

Dysregulation of MUC19 was found to be strongly correlated with the progression of breast cancer. A previous study disclosed that MUC19 level was elevated in breast cancer. Moreover, Yang et al*.* found that downregulation of MUC19 restrained cell proliferation and colony formation of BC cells. To deeply explore the mechanism of MUC19 in BC cells, Targetscan database was used to predict the downstream targets of miR-1287-5p, and dual-luciferase reporter assay verified the binding sites between miR-1287-5p and MUC19. MUC19 was notably upregulated in BC tissues and cells. In addition, MUC19 was negatively regulated by miR-1287-5p in BC cells. Rescue experiments revealed that overexpression of MUC19 overturned the effects of miR-1287-5p mimic on BC cells, suggesting that miR-1287-5p restrained BC progression by downregulating MUC19. Circ_0001982 elevated MUC19 expression by sponging miR-1287-5p in BC cells. To explore the effect of circ_0001982 knockdown on tumor development in vivo, the xenograft tumor model was used. And the results suggested that circ_0001982 knockdown could inhibit xenograft tumor growth in vivo.

There are also some limitations in our studies. The in vivo function of circ_0001982 in regulating tumor metastasis needs to be explored through the xenograft tumor model. In addition, other miRNA and mRNA targets of circ_0001982 need to be sought to further illustrate the regulatory mechanism of circ_0001982 in BC progression. In the future, we will determine the level of circ_0001982 in exosomal to analyze if circ_0001982 could be used as a novel blood biomarker for the early diagnosis of BC. Despite the above limitations, our study illustrated the regulatory function of circ_0001982/miR-1287-5p/MUC19 axis in regulating the glycolysis, cell viability, and motility of BC cells. Meanwhile, this is the first study showing the interaction between miR-1287-5p and circ_0001982 or MUC19.

In conclusion, circ_0001982 contributed to the glycolytic metabolism, cell viability, migration, and invasion of BC cells via regulating miR-1287-5p/MUC19 axis. Circ_0001982/miR-1287-5p/MUC19 axis might be a novel potential target for BC treatment.

## Data Availability

The datasets used and/or analyzed during the current study are available from the corresponding author on reasonable request.
